# Supplementary material for: Consequences of Autophagy Deletion on the Age-Related Changes in the Epidermal Lipidome of Mice
Source: Int J Mol Sci. 2022 Sep 21;23(19):11110. doi: 10.3390/ijms231911110 (PMC9569666; doi:10.3390/ijms231911110)
Supplement: Supplementary file 1 [file ijms-23-11110-s001.zip › ijms-1854409-supplementary.pdf]

Supplementary Material

	Mean (pmol/mg wet epidermis)				SD				t-test	
	Atg7$^{F/F}$ young	*Atg7*$^{F/F}$ old	*atg7ΔKC* young	*atg7ΔKC* old	*Atg7*$^{F/F}$ young	*Atg7*$^{F/F}$ old	*atg7ΔKC* young	*atg7ΔKC* old	*Atg7*$^{F/F}$ old vs young	Old *atg7ΔKC* vs *Atg7*$^{F/F}$
**TAG 44:0**		10.4033		24.3734		1.97		27.94	-	ns
**TAG 44:1**	13.5501	36.2795	13.3397	35.9195	2.89	6.86	6.04	25.31	**	ns
**TAG 44:2**	16.8223	46.3396	16.0590	35.6732	2.86	7.38	7.23	28.13	**	ns
**TAG 46:0**	15.1837	18.8238	15.9314	31.1501	1.48	3.60	2.40	29.10	ns	ns
**TAG 46:1**	68.1928	164.7452	57.3917	90.7035	11.56	20.46	25.99	80.43	**	ns
**TAG 46:2**	95.4160	343.3570	98.6145	200.5855	14.58	59.89	49.31	189.75	**	ns
**TAG 46:4**	93.5555	72.1946	81.0361	64.2545	15.93	13.24	15.03	28.89	ns	ns
**TAG 48:0**	17.9693	25.2074	19.1237	23.2413	1.82	4.62	4.64	17.41	*	ns
**TAG 48:1**	127.1839	377.6745	132.0091	166.2450	25.25	75.82	51.63	138.16	**	*
**TAG 48:2**	495.2667	1634.1266	460.2430	821.6008	76.18	291.95	233.62	770.52	**	0.08872
**TAG 48:3**	359.7180	1466.2676	375.8811	823.7745	66.19	268.49	196.78	794.56	**	ns
**TAG 49:1**	16.8670	39.4612	19.2426	23.9257	1.90	3.61	6.51	14.98	**	0.08535
**TAG 49:2**	87.9940	159.8873	95.8149	117.6468	6.01	26.00	20.31	55.27	**	ns
**TAG 49:3**	44.3383	157.5726	51.6250	87.1399	9.10	26.97	26.14	74.11	**	ns
**TAG 50:1**	156.5134	347.3253	158.1194	155.9763	40.01	52.44	73.41	120.96	**	*
**TAG 50:2**	859.8766	3156.5263	895.3974	1263.3986	158.33	257.40	415.03	1113.14	**	*
**TAG 50:3**	1615.1374	6394.0110	1547.6267	3026.2684	343.24	1065.04	832.16	2887.53	**	0.06459
**TAG 50:4**	585.5558	2594.1986	613.7884	1279.3798	168.25	404.17	321.17	1244.61	**	0.08493
**TAG 51:1**		12.8201		9.4444		2.12		3.98	-	ns
**TAG 51:2**	40.6591	147.5657	50.6998	73.6563	7.10	13.41	23.92	52.25	**	*
**TAG 51:3**	70.4383	257.8790	82.3350	138.9240	17.22	47.26	42.00	110.79	**	0.08738
**TAG 51:4**	50.4269	158.9171	59.6725	83.2883	11.98	34.28	28.87	63.87	**	0.07199
**TAG 52:1**	18.7090	21.8895	19.7481	16.0173	2.33	4.26	6.21	6.63	ns	ns
**TAG 52:2**	664.5318	2364.8289	724.7074	964.9028	195.78	645.52	395.07	924.81	**	*
**TAG 52:3**	1908.9731	8854.4262	2089.2756	3946.7149	441.92	1532.10	1101.70	3658.05	**	*
**TAG 52:4**	2085.0253	7934.1433	1990.9444	3498.4761	556.93	2131.18	1038.32	3176.92	**	*
**TAG 53:2**	14.1109	35.9396	17.0551	19.4784	1.59	6.09	6.54	10.74	**	*
**TAG 53:3**	44.1513	174.5117	53.2630	83.0388	8.70	21.65	24.92	62.73	**	*
**TAG 53:4**	35.3965	130.4304	41.1470	65.9123	11.95	29.02	22.20	51.48	**	0.06179
**TAG 54:2**	38.3435	71.5107	40.5084	40.6765	8.06	18.95	15.80	27.08	*	ns
**TAG 54:3**	836.1399	5062.1274	1026.9962	2161.9761	246.42	2150.42	602.21	2029.64	**	0.07651
**TAG 54:4**	1402.0929	6135.3807	1507.1070	2352.1073	419.01	1733.03	827.08	2018.39	**	*
**TAG 54:5**	982.6527	3043.2537	927.9104	1225.7940	287.81	957.81	500.49	1029.94	**	*
**TAG 54:6**	397.6577	1006.1942	406.9454	491.5107	96.26	258.18	165.43	387.59	**	0.05762
**TAG 55:3**		22.6810	17.6509	12.4518		2.21		5.95	-	*
**TAG 56:4**	42.0974	111.4824	46.0092	47.6437	10.99	13.41	19.37	29.37	**	*
**TAG 56:7**	24.4377	105.4737	26.7742	45.7712	5.63	39.27	16.03	41.88	*	0.08277
**TAG 58:8**	54.4140	331.1916	60.4801	114.9423	17.23	141.31	28.26	115.14	**	*
**TAG 58:9**	33.6351	237.2813	42.5665	78.4360	10.12	107.33	22.91	80.58	**	*

Supplementary Table S1: Most of the triacylglycerol (TAG) species contribute to the increased total TAG amount found in the aged groups, especially in *Atg7*$^{F/F}$ old mice.

Identified TAG species and their means and standard deviations (SD) of absolute amount analysed in epidermal extracts (pmol/mg wet epidermis). Together were identified 39 TAG species, 3 of them were detected only in the aged groups (TAG 44:0, 51:1, 55:3). None (0/36) were significantly regulated in *atg7*ΔKC young, 33/36 species were upregulated in *Atg7*$^{F/F}$ old compared to the control - *Atg7*$^{F/F}$ young, respectively. Non-regulated in the aged control group are TAG 46:0, 46:4, 52:1. 34/36 species were decreased (not all significantly, due to the big SD) in the aged knockouts compared to the aged controls. Comparing aged vs. young *atg7*ΔKC no significant differences were found;

Student´s t-test, *p < 0,05; **p < 0,01; n = 4 (for Atg7f/f young and old, and atg7ΔKC young) and 5 (for atg7ΔKC old)

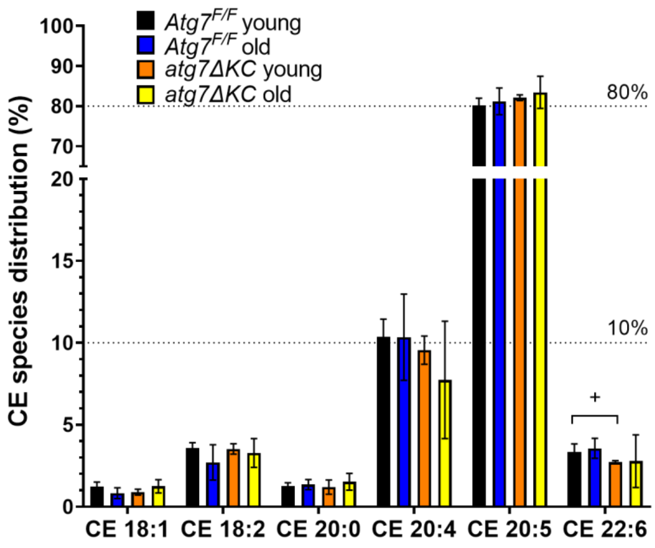

Supplementary Figure S1: The proportional distribution of identified cholesteryl ester (CE) species in the studied groups. Statistic: Student´s t-test, * marks a significant difference compared to the reference group, + shows a significant difference between two respective groups indicated by a line segment; */+p < 0,05, **/++p < 0,01; n = 4 (for Atg7f/f young and old, and atg7ΔKC young) and 5 (for atg7ΔKC old).

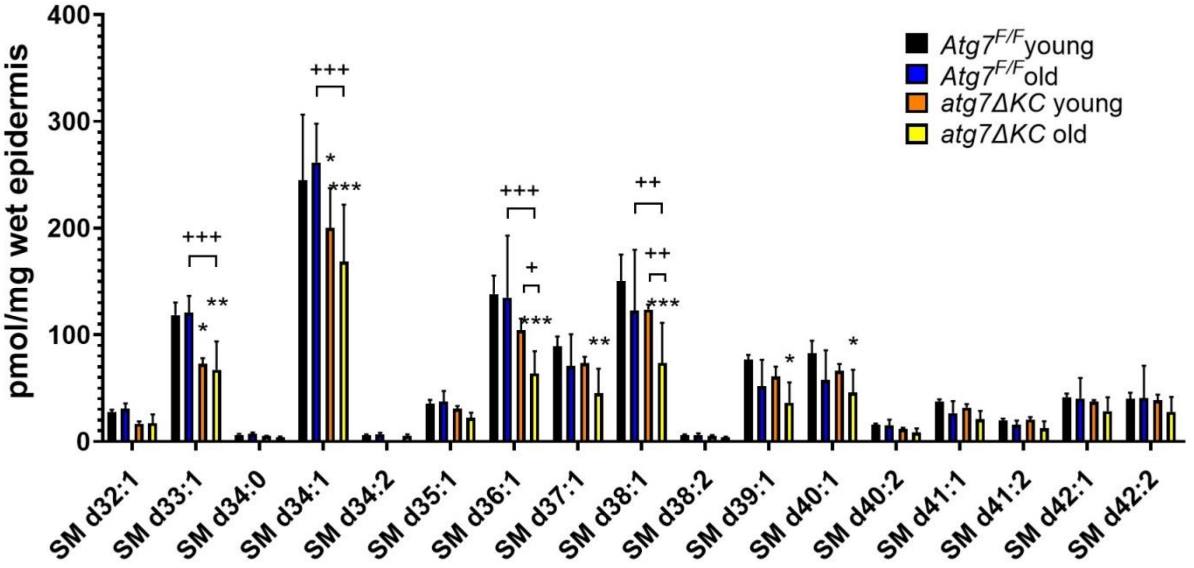

Supplementary Figure S2: Short acyl-chain sphingomyelins (SM) are significantly age-independently decreased in knockout phenotype, whereas levels of long acyl-chain SM are significantly reduced only in aged knockout group.

Overview of all identified sphingomyelin (SM) species. Absolut amounts are expressed as pmol/mg of hydrated epidermal tissue. Two-way ANOVA with Bonferroni's multiple comparisons test, mean ± standard deviation. * marks a significant difference compared to the control group (*Atg7*$^{F/F}$ young), **+** shows a significant difference between two respective groups indicated by a line segment; */+p < 0,05, **/++p < 0,01, ***/+++p < 0,001; n = 4 (for Atg7f/f young and old, and atg7ΔKC young) and 5 (for atg7ΔKC old). Short acyl-chain SM: 32-35C, long chain-acyl SM: 36-42C

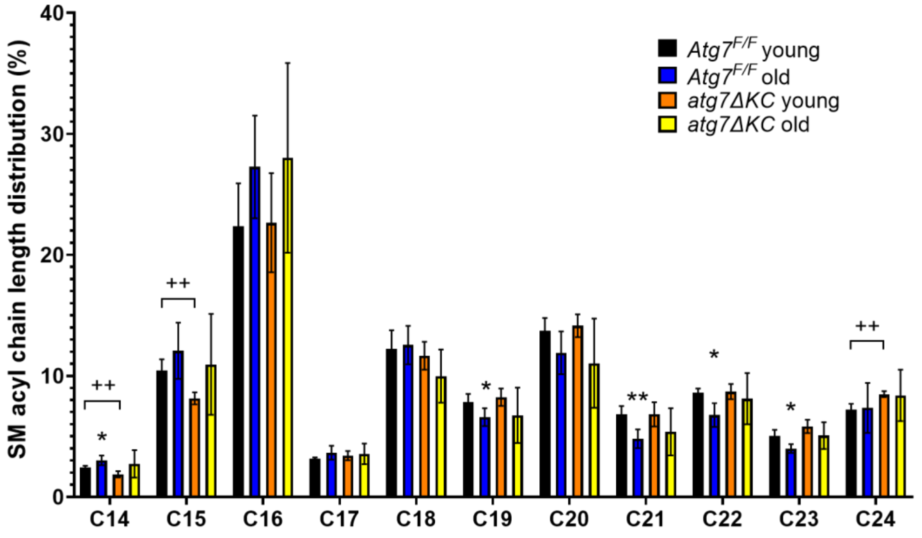

Supplementary Figure S3: The proportion profile of predicted acyl-chain lengths within the sphingomyelin (SM) class in the studied groups. SM with the identical carbon number are combined (assuming that the sphingoid base has 18C, e.g. predicted C16 = SM d34:x). Statistic: Student´s t-test, * marks a significant difference compared to the reference group, + shows a significant difference between two respective groups indicated by a line segment; */+p < 0,05, **/++p < 0,01; n = 4 (for Atg7f/f young and old, and atg7ΔKC young) and 5 (for atg7ΔKC old).

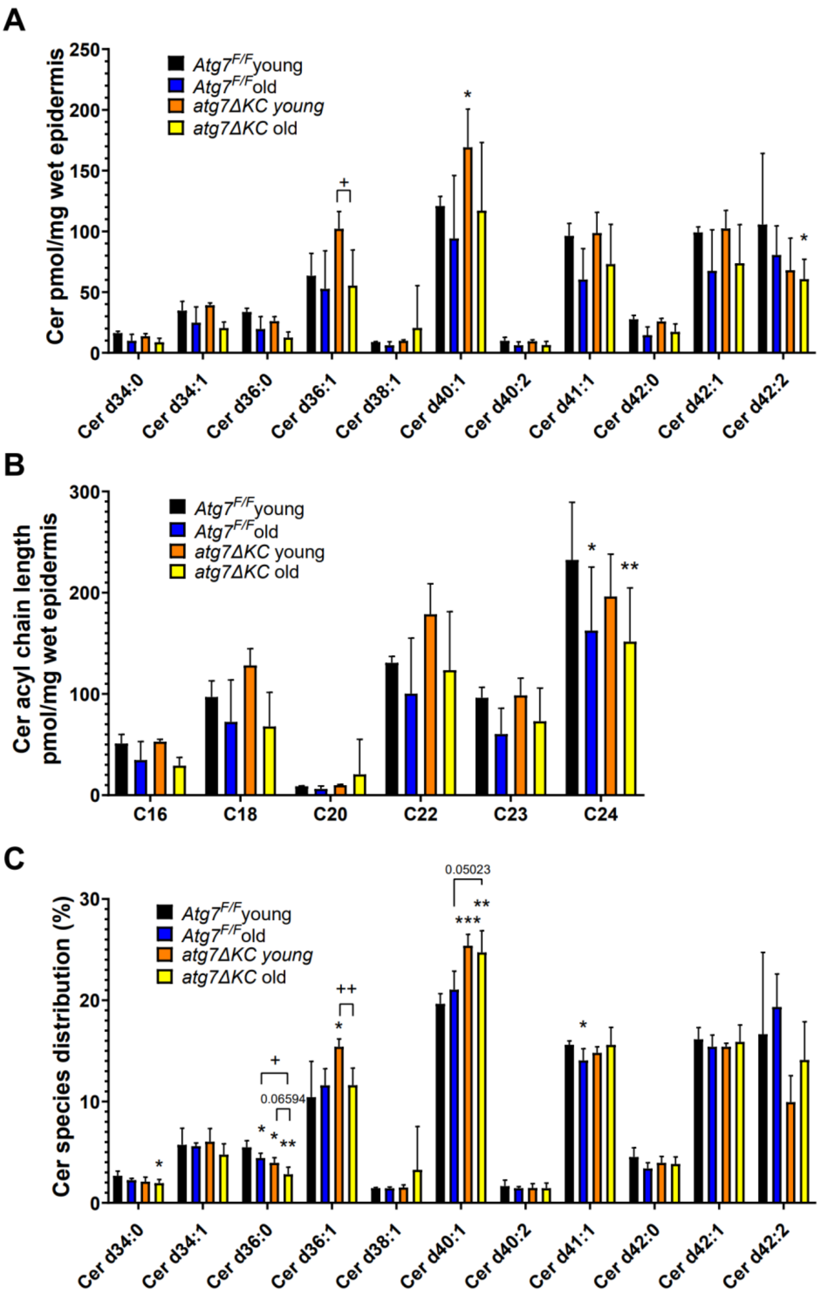

Supplementary Figure S4: Profile of ceramides (Cer) identified in murine epidermal extracts.

A Absolute amounts are expressed as pmol/mg of hydrated epidermal tissue. Two-way ANOVA with Bonferroni's multiple comparisons test, mean ± standard deviation. * marks a significant difference compared to the control group ($Atg7^{F/F}$ young), + shows a significant difference between two respective groups indicated by a line segment; */+p < 0,05, **/++p < 0,01; n = 4 (for Atg7f/f young and old, and atg7ΔKC young) and 5 (for atg7ΔKC old). **B** Cer with the identical carbon number are combined (assuming that the sphingoid base has 18C, e.g. predicted C16 = Cer d34:x). Absolute amounts are expressed as pmol/mg of hydrated epidermal tissue. Two-way ANOVA with Bonferroni's multiple comparisons test, mean ± standard deviation. * marks a significant difference compared to the control group ($Atg7^{F/F}$ young, * < 0,05, **p < 0,01; n = 4 (for Atg7f/f young and old, and atg7ΔKC young) and 5 (for atg7ΔKC old). **C** The proportional distribution of individual identified species within the Cer class. Student´s t-test, * marks a significant difference compared to the reference group, + shows a significant difference between two respective groups indicated by a line segment; */+p <

0,05, **/++$p < 0,01$, ***/+++$p < 0,001$; $n = 4$ (for Atg7f/f young and old, and atg7ΔKC young) and 5 (for atg7ΔKC old).
